# Supplementary material for: Identification of phosphatases that dephosphorylate the co-chaperone BAG3
Source: Life Sci Alliance. 2024 Nov 19;8(2):e202402734. doi: 10.26508/lsa.202402734 (PMC11576475; doi:10.26508/lsa.202402734)

Figure 2 A

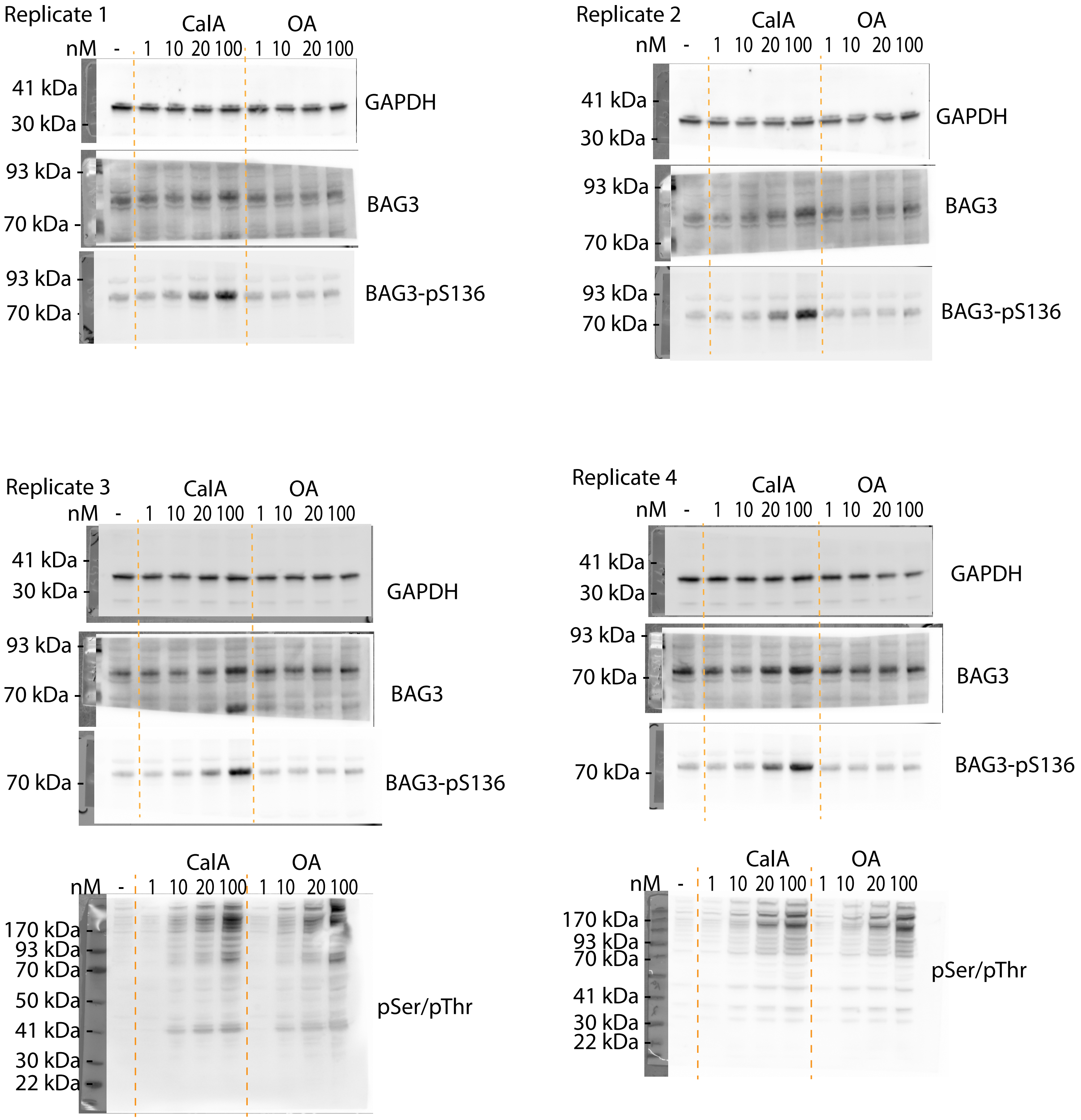
Replicate 1
CalA
OA
nM - 1 10 20 100 1 10 20 100
41 kDa
30 kDa
GAPDH
93 kDa
70 kDa
BAG3
93 kDa
70 kDa
BAG3-pS136
Replicate 2
CalA
OA
nM - 1 10 20 100 1 10 20 100
41 kDa
30 kDa
GAPDH
93 kDa
70 kDa
BAG3
93 kDa
70 kDa
BAG3-pS136
Replicate 3
CalA
OA
nM - 1 10 20 100 1 10 20 100
41 kDa
30 kDa
GAPDH
93 kDa
70 kDa
BAG3
70 kDa
BAG3-pS136
Replicate 4
CalA
OA
nM - 1 10 20 100 1 10 20 100
41 kDa
30 kDa
GAPDH
93 kDa
70 kDa
BAG3
70 kDa
BAG3-pS136
CalA
OA
nM - 1 10 20 100 1 10 20 100
170 kDa
93 kDa
70 kDa
50 kDa
41 kDa
30 kDa
22 kDa
pSer/pThr
CalA
OA
nM - 1 10 20 100 1 10 20 100
170 kDa
93 kDa
70 kDa
41 kDa
30 kDa
22 kDa
pSer/pThr

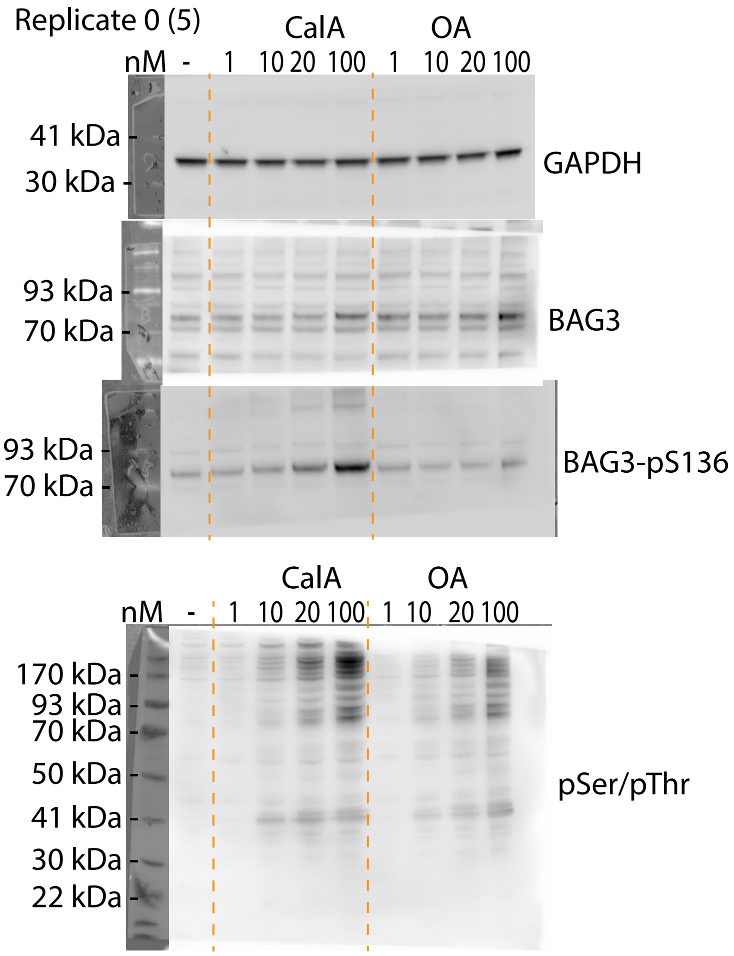
Replicate 0 (5)
CalA
OA
nM - 1 10 20 100 1 10 20 100
41 kDa
30 kDa
GAPDH
93 kDa
70 kDa
BAG3
93 kDa
70 kDa
BAG3-pS136
CalA
OA
nM - 1 10 20 100 1 10 20 100
170 kDa
93 kDa
70 kDa
50 kDa
41 kDa
30 kDa
22 kDa
pSer/pThr

Figure 2 D

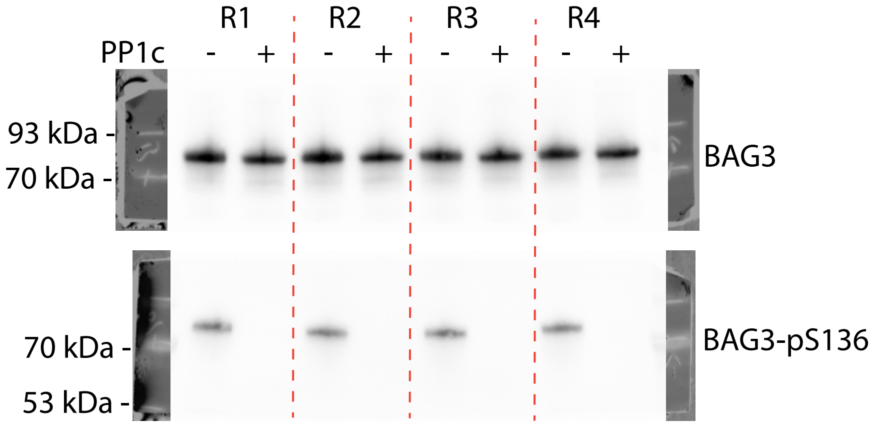
R1
R2
R3
R4
PP1c - + - + - + - +
93 kDa
70 kDa
BAG3
70 kDa
53 kDa
BAG3-pS136

Figure 2 E

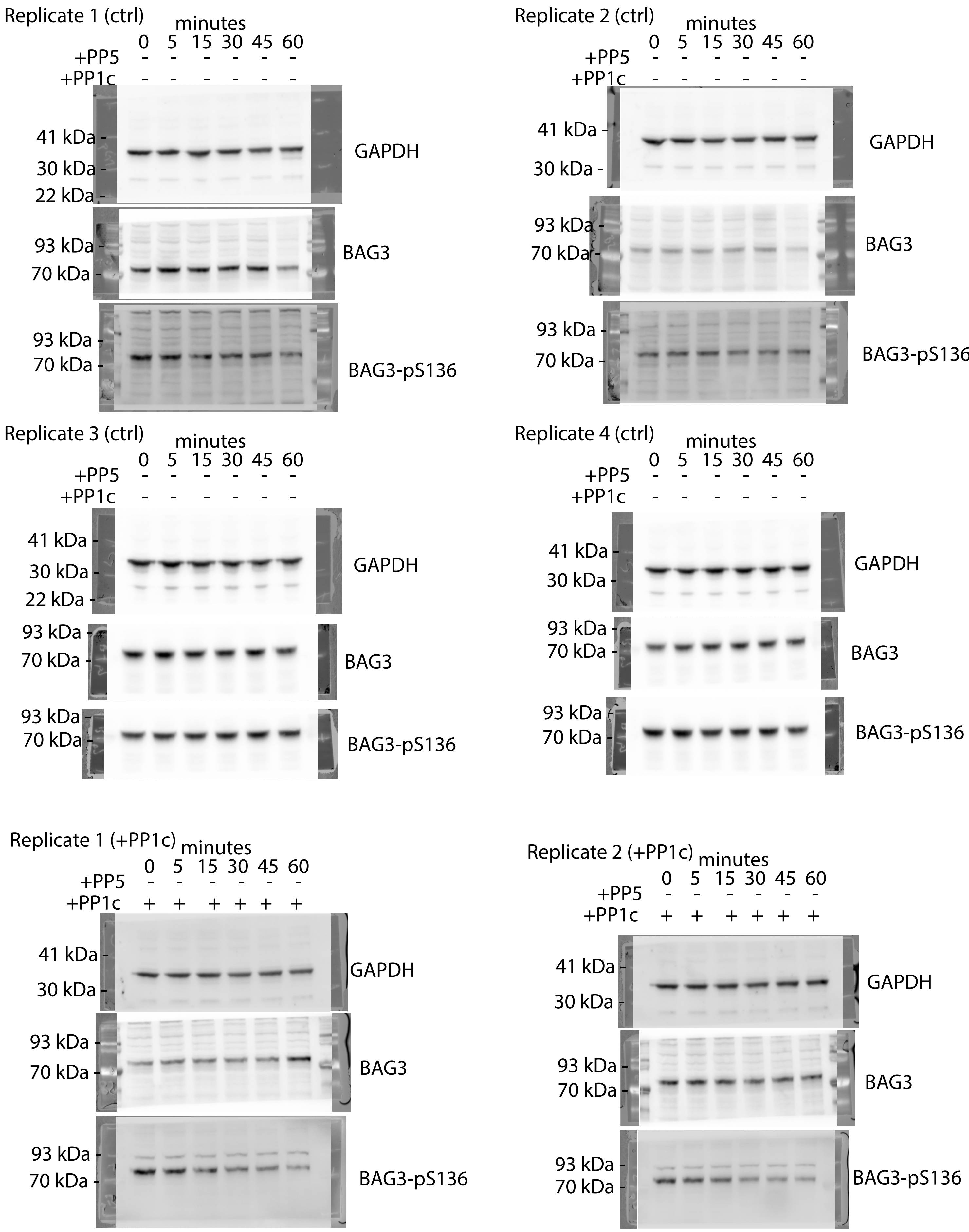
Replicate 1 (ctrl)
minutes
0 5 15 30 45 60
+PP5 - - - - - -
+PP1c - - - - - -
41 kDa
30 kDa
22 kDa
GAPDH
93 kDa
70 kDa
BAG3
93 kDa
70 kDa
BAG3-pS136
Replicate 2 (ctrl)
minutes
0 5 15 30 45 60
+PP5 - - - - - -
+PP1c - - - - - -
41 kDa
30 kDa
GAPDH
93 kDa
70 kDa
BAG3
93 kDa
70 kDa
BAG3-pS136
Replicate 3 (ctrl)
minutes
0 5 15 30 45 60
+PP5 - - - - - -
+PP1c - - - - - -
41 kDa
30 kDa
22 kDa
GAPDH
93 kDa
70 kDa
BAG3
93 kDa
70 kDa
BAG3-pS136
Replicate 4 (ctrl)
minutes
0 5 15 30 45 60
+PP5 - - - - - -
+PP1c - - - - - -
41 kDa
30 kDa
GAPDH
93 kDa
70 kDa
BAG3
93 kDa
70 kDa
BAG3-pS136
Replicate 1 (+PP1c)
minutes
0 5 15 30 45 60
+PP5 - - - - - -
+PP1c + + + + + +
41 kDa
30 kDa
GAPDH
93 kDa
70 kDa
BAG3
93 kDa
70 kDa
BAG3-pS136
Replicate 2 (+PP1c)
minutes
0 5 15 30 45 60
+PP5 - - - - - -
+PP1c + + + + + +
41 kDa
30 kDa
GAPDH
93 kDa
70 kDa
BAG3
93 kDa
70 kDa
BAG3-pS136

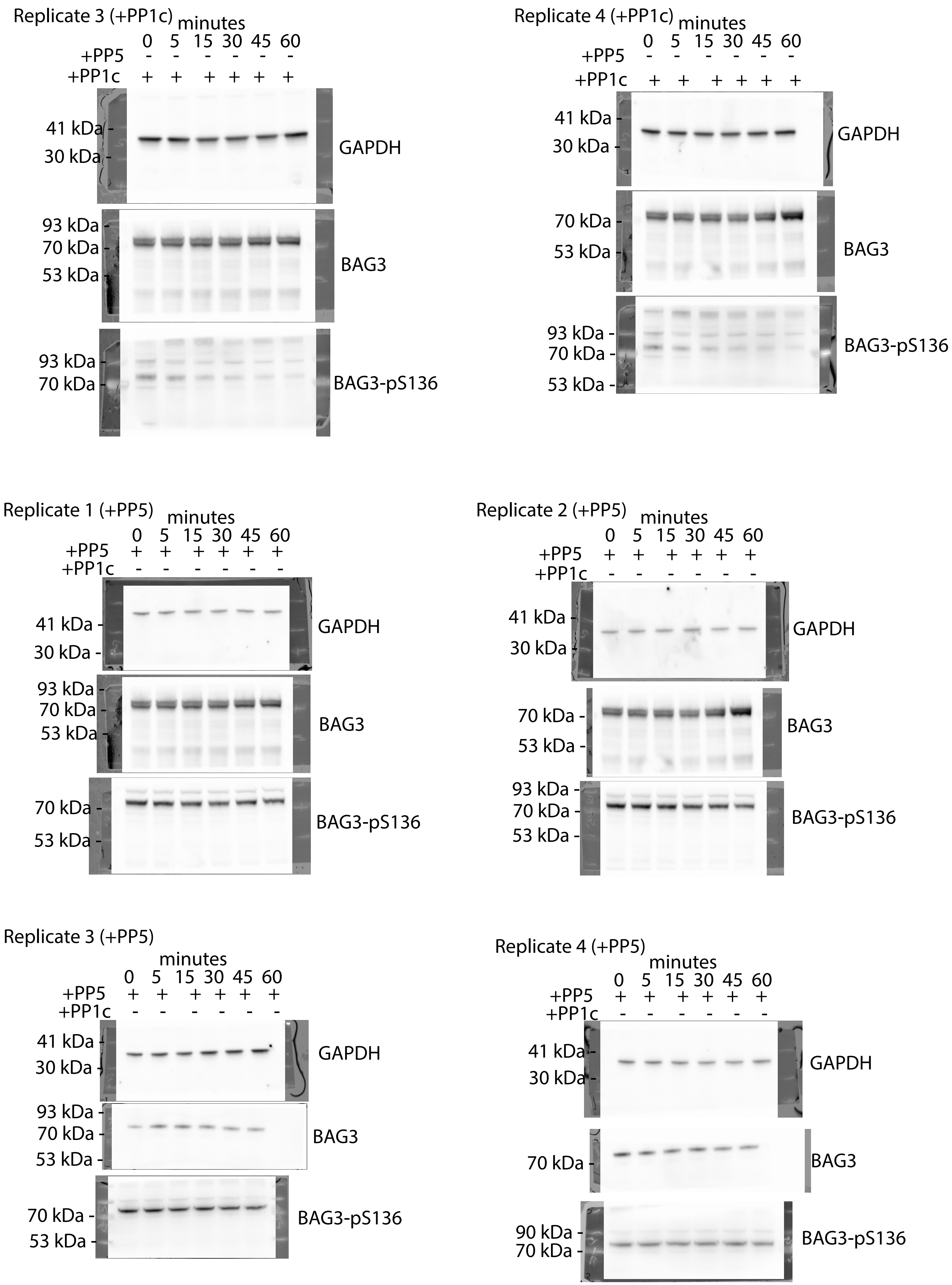
Replicate 3 (+PP1c)
minutes
0 5 15 30 45 60
+PP5 - - - - - -
+PP1c + + + + + +
41 kDa
30 kDa
GAPDH
93 kDa
70 kDa
53 kDa
BAG3
93 kDa
70 kDa
BAG3-pS136
Replicate 4 (+PP1c)
minutes
0 5 15 30 45 60
+PP5 - - - - - -
+PP1c + + + + + +
41 kDa
30 kDa
GAPDH
70 kDa
53 kDa
BAG3
93 kDa
70 kDa
53 kDa
BAG3-pS136
Replicate 1 (+PP5)
minutes
0 5 15 30 45 60
+PP5 + + + + + +
+PP1c - - - - - -
41 kDa
30 kDa
GAPDH
93 kDa
70 kDa
53 kDa
BAG3
70 kDa
53 kDa
BAG3-pS136
Replicate 2 (+PP5)
minutes
0 5 15 30 45 60
+PP5 + + + + + +
+PP1c - - - - - -
41 kDa
30 kDa
GAPDH
70 kDa
53 kDa
BAG3
93 kDa
70 kDa
53 kDa
BAG3-pS136
Replicate 3 (+PP5)
minutes
0 5 15 30 45 60
+PP5 + + + + + +
+PP1c - - - - - -
41 kDa
30 kDa
GAPDH
93 kDa
70 kDa
53 kDa
BAG3
70 kDa
53 kDa
BAG3-pS136
Replicate 4 (+PP5)
minutes
0 5 15 30 45 60
+PP5 + + + + + +
+PP1c - - - - - -
41 kDa
30 kDa
GAPDH
70 kDa
BAG3
90 kDa
70 kDa
BAG3-pS136

Supplementary Figure 2 A

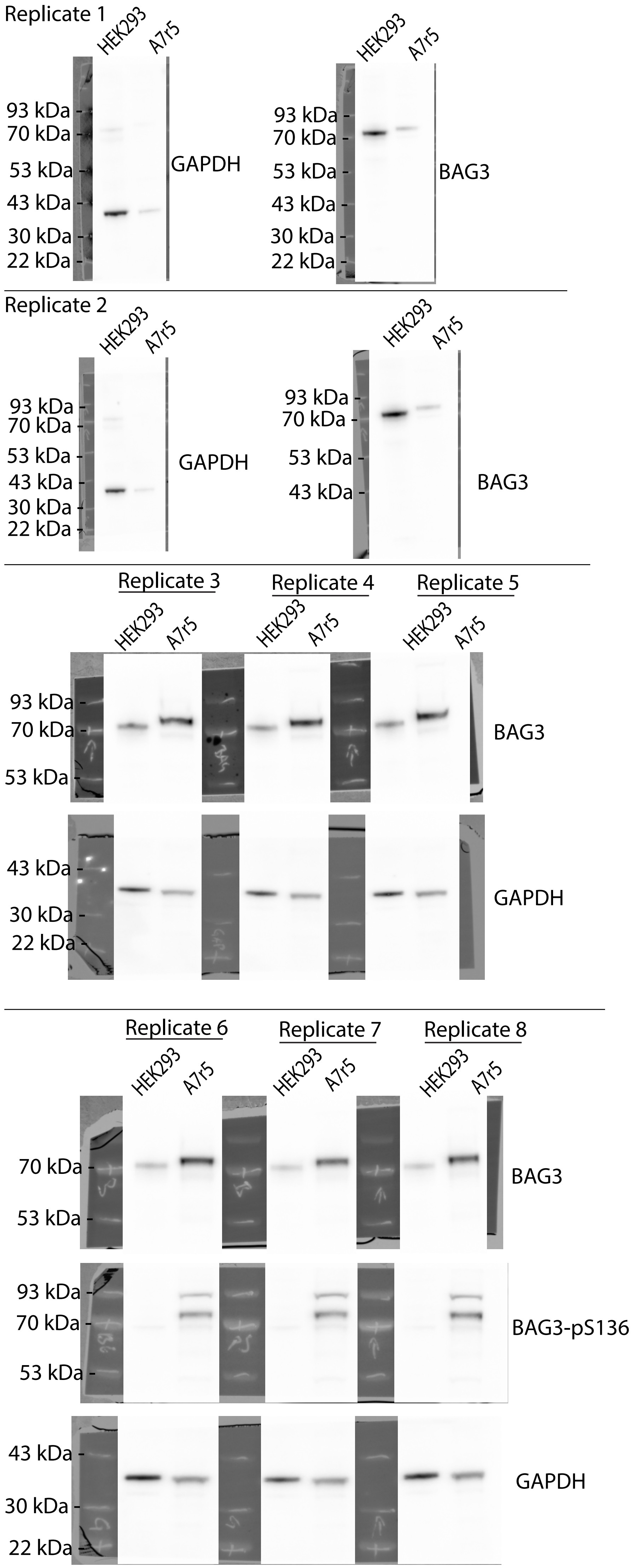

Supplementary Figure 2 D

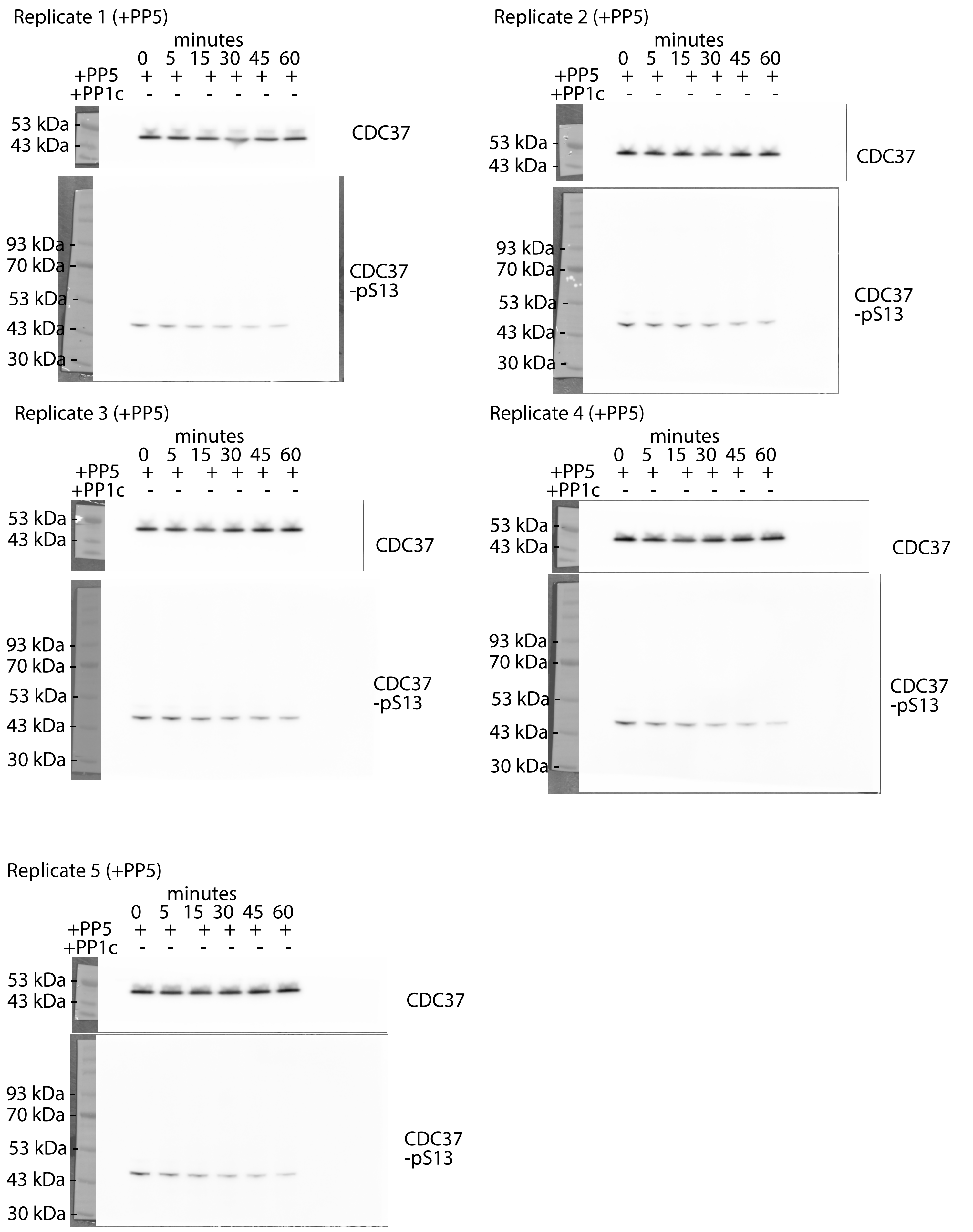

Supplement: Supplementary file 2 [file LSA-2024-02734_SdataF2.1_FS2.pdf]
