# Supplementary material for: Identification of phosphatases that dephosphorylate the co-chaperone BAG3
Source: Life Sci Alliance. 2024 Nov 19;8(2):e202402734. doi: 10.26508/lsa.202402734 (PMC11576475; doi:10.26508/lsa.202402734)

Figure 3 A

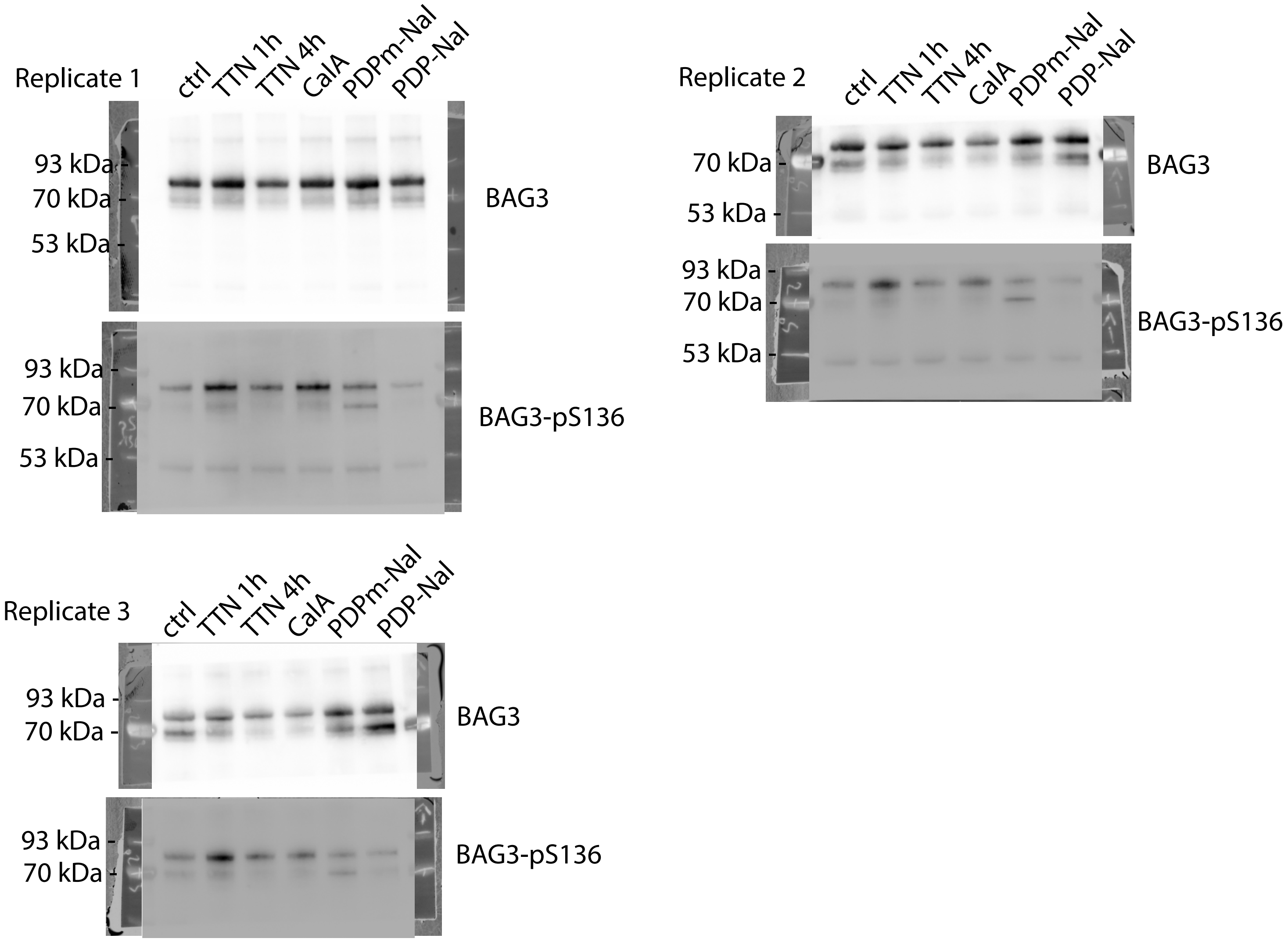

Figure 3 B

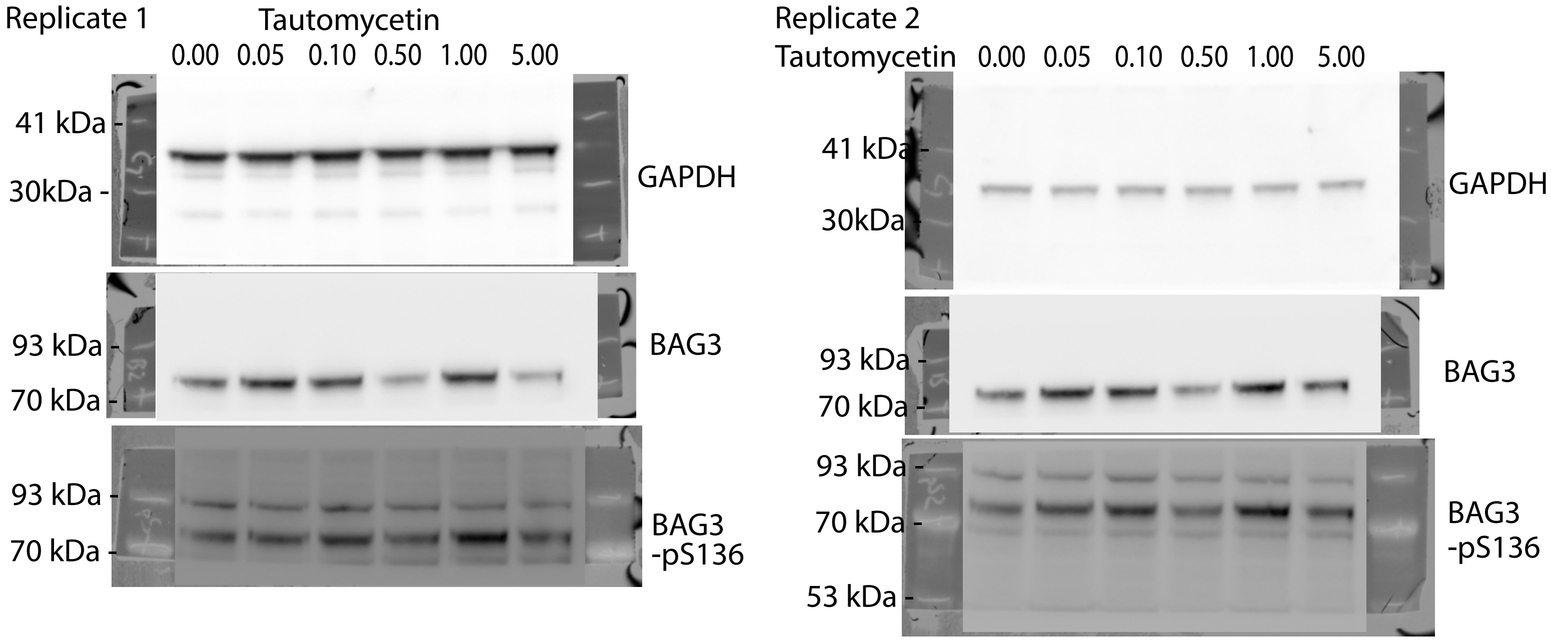

Replicate 3

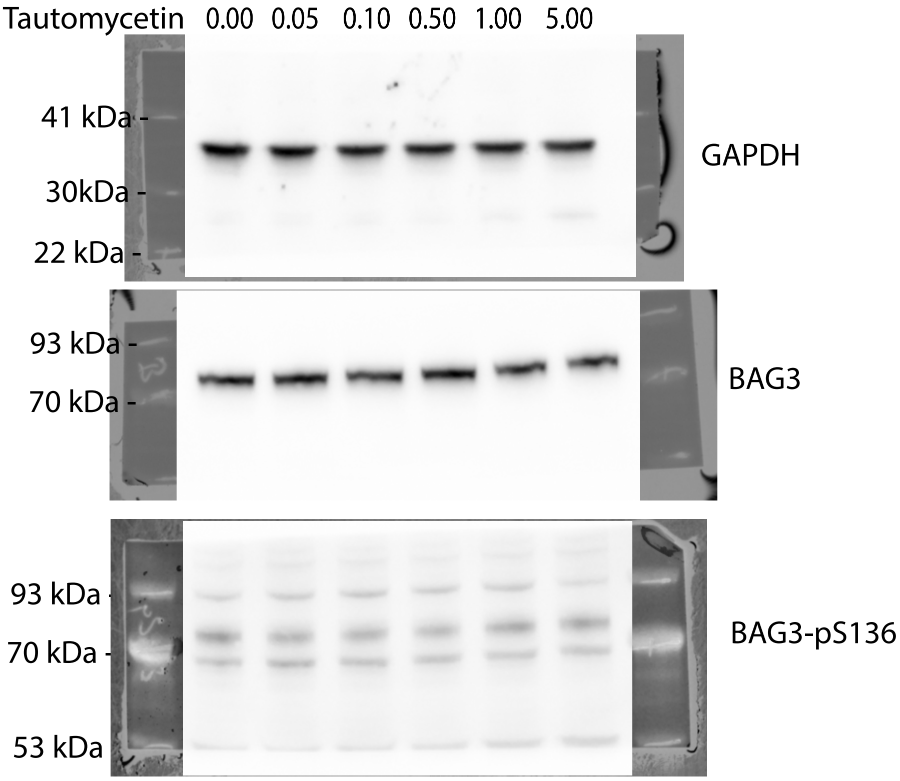

Replicate 4

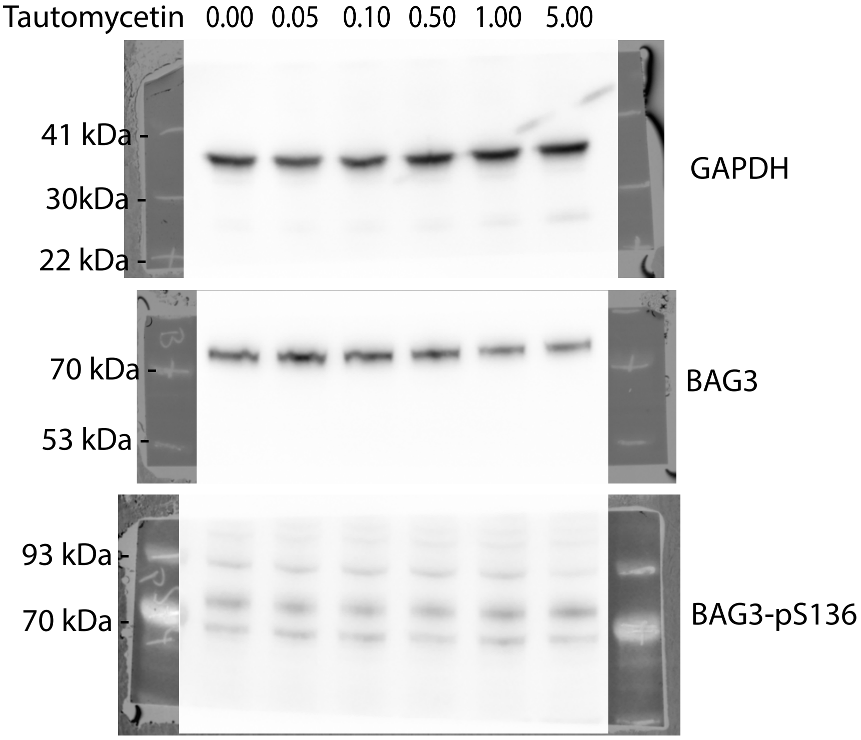

Figure 3 C

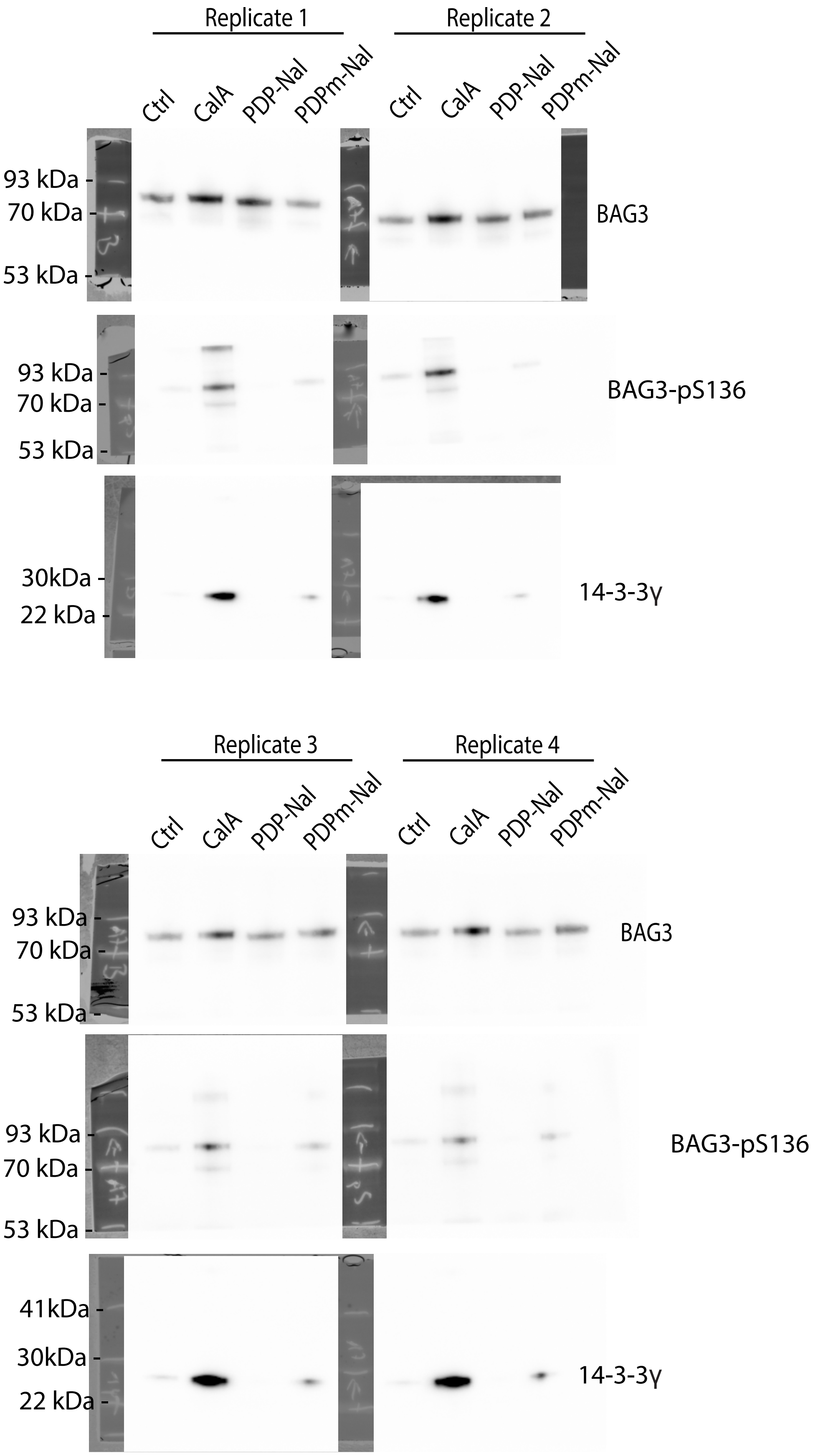

Replicate 1
Replicate 2
Ctrl
CalA
PDP-Nal
PDPm-Nal
Ctrl
CalA
PDP-Nal
PDPm-Nal
93 kDa
70 kDa
53 kDa
BAG3
93 kDa
70 kDa
53 kDa
BAG3-pS136
30kDa
22 kDa
14-3-3γ
Replicate 3
Replicate 4
Ctrl
CalA
PDP-Nal
PDPm-Nal
Ctrl
CalA
PDP-Nal
PDPm-Nal
93 kDa
70 kDa
53 kDa
BAG3
93 kDa
70 kDa
53 kDa
BAG3-pS136
41kDa
30kDa
22 kDa
14-3-3γ

Figure 3 D - PP1CA/B/C

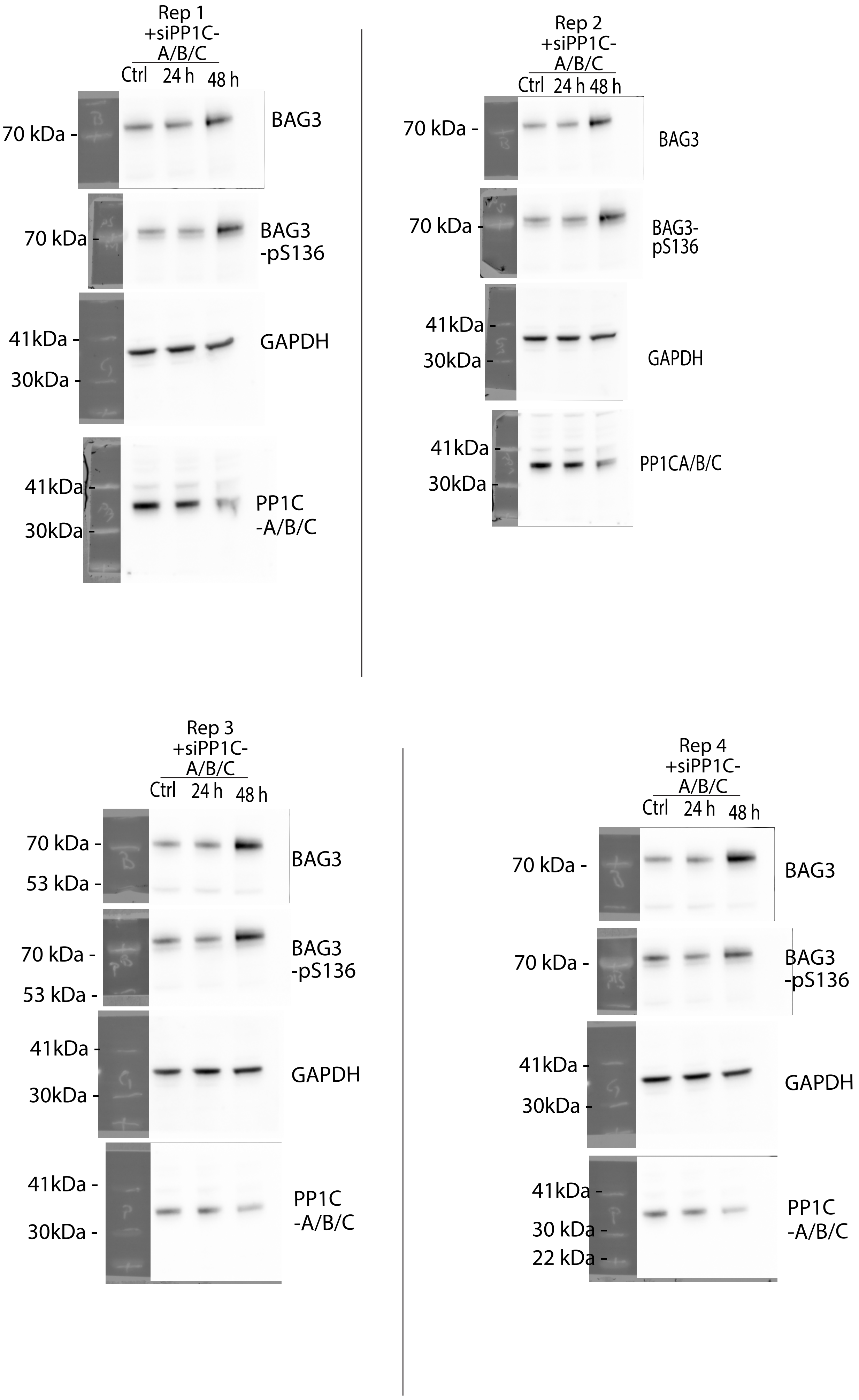

Figure 3 D - PP1CA

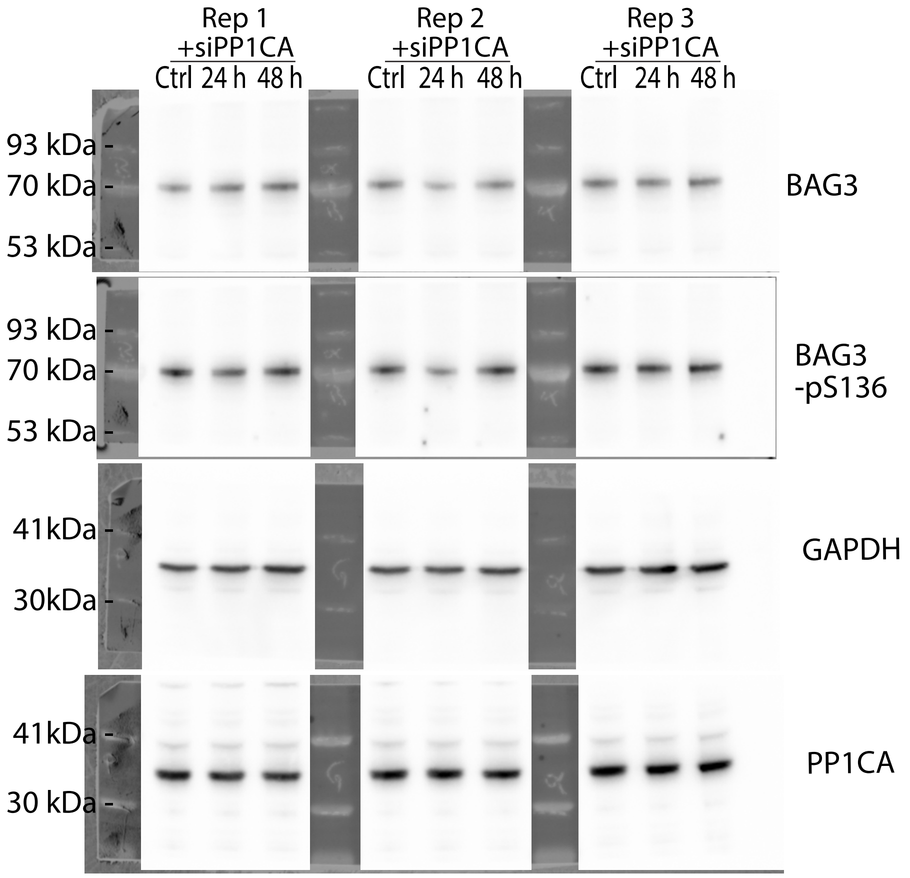

Figure 3 D - PP1CB

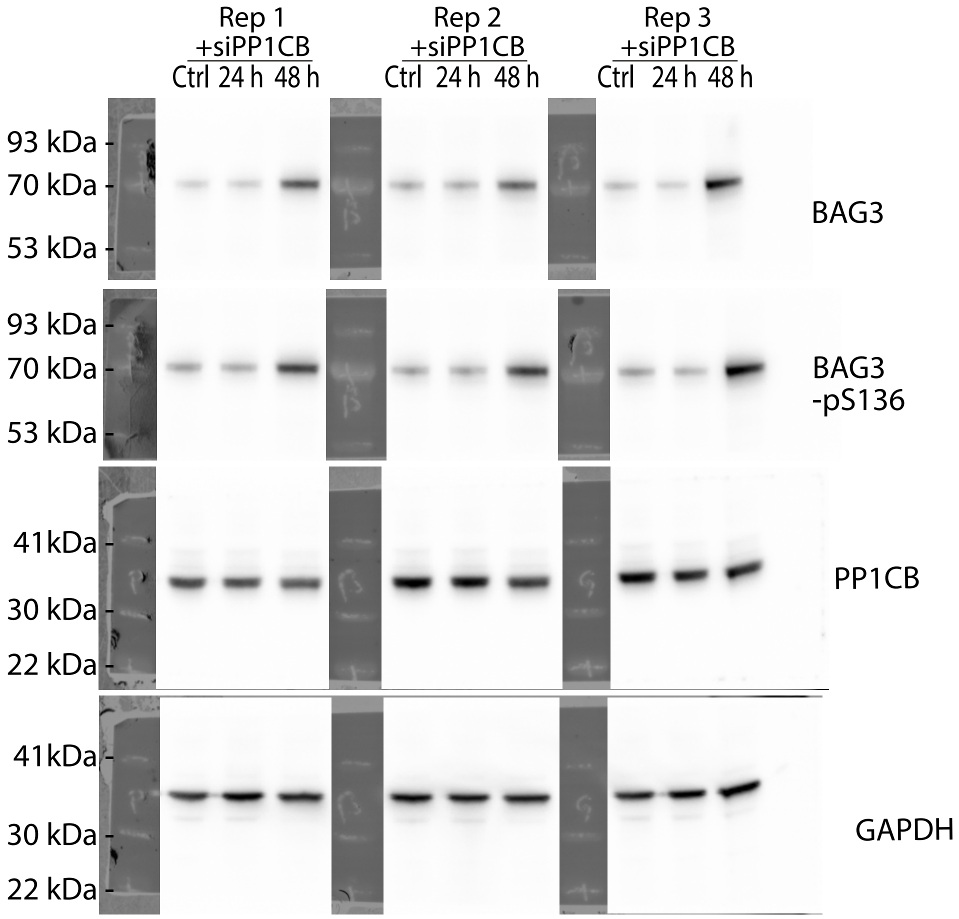

Figure 3 D - PP1CC

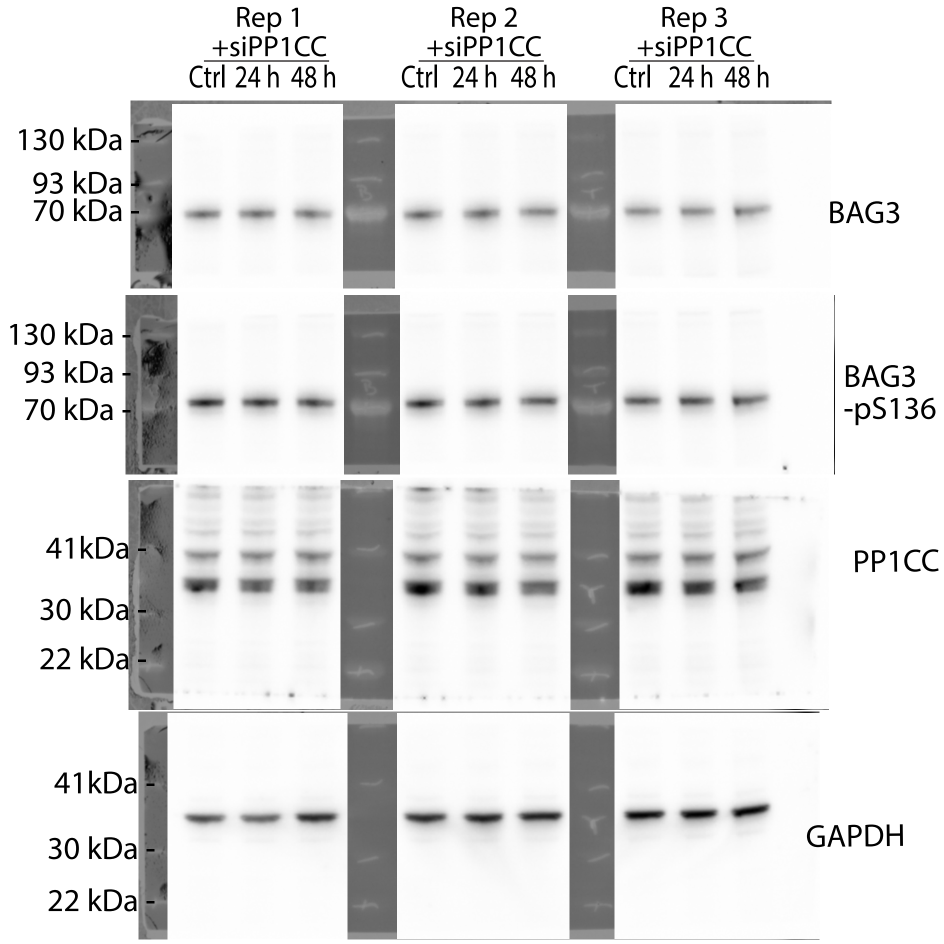

Supplement: Supplementary file 4 [file LSA-2024-02734_SdataF3_FS3.pdf]
