# Supplementary material for: Identification of phosphatases that dephosphorylate the co-chaperone BAG3
Source: Life Sci Alliance. 2024 Nov 19;8(2):e202402734. doi: 10.26508/lsa.202402734 (PMC11576475; doi:10.26508/lsa.202402734)

Figure 5 A

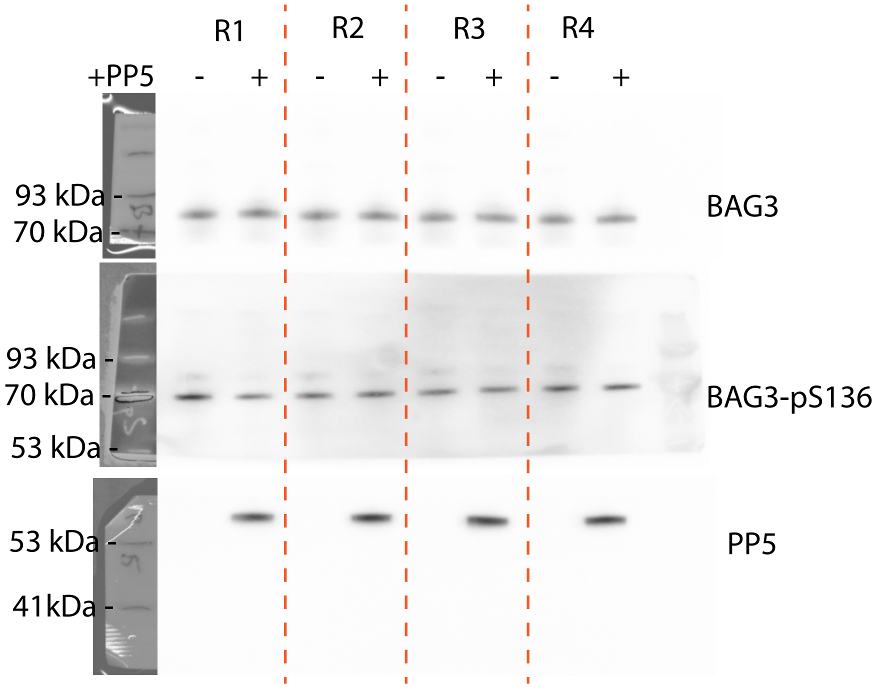

R1
R2
R3
R4
+PP5
-
+
-
+
-
+
-
+
93 kDa -
70 kDa -
BAG3
93 kDa -
70 kDa -
53 kDa -
BAG3-pS136
53 kDa -
41kDa -
PP5

Figure 5C

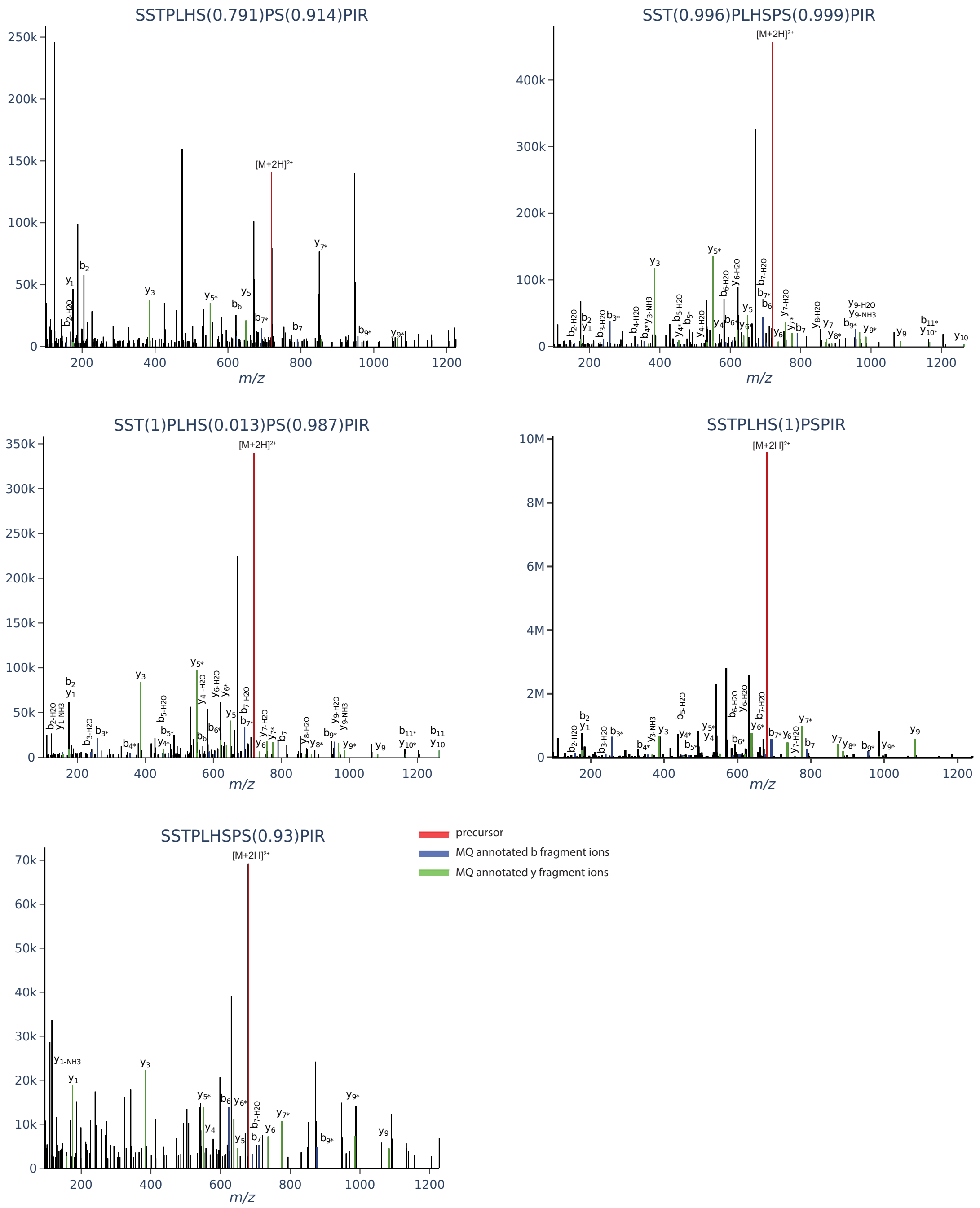

SSTPLHS(0.791)PS(0.914)PIR
SST(0.996)PLHSPS(0.999)PIR
SST(1)PLHS(0.013)PS(0.987)PIR
SSTPLHS(1)PSPIR
SSTPLHSPS(0.93)PIR
[M+2H]2+
m/z
precursor
MQ annotated b fragment ions
MQ annotated y fragment ions

Figure 5E/ Supplementary Figure 5 D

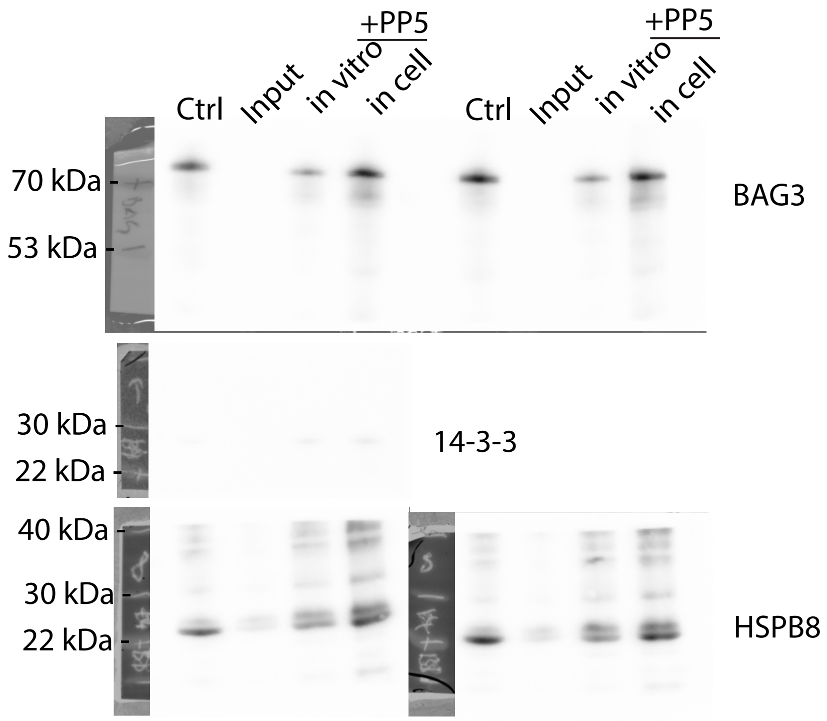

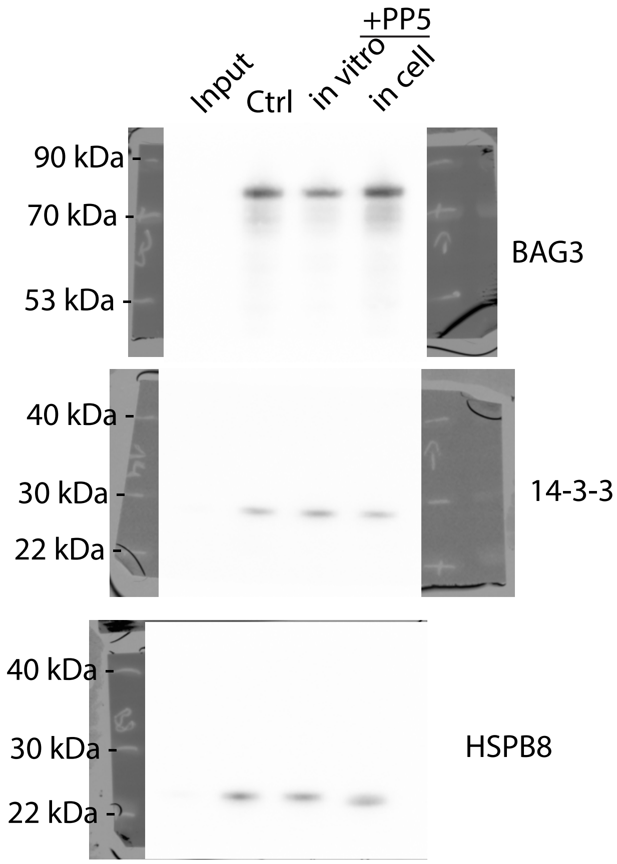

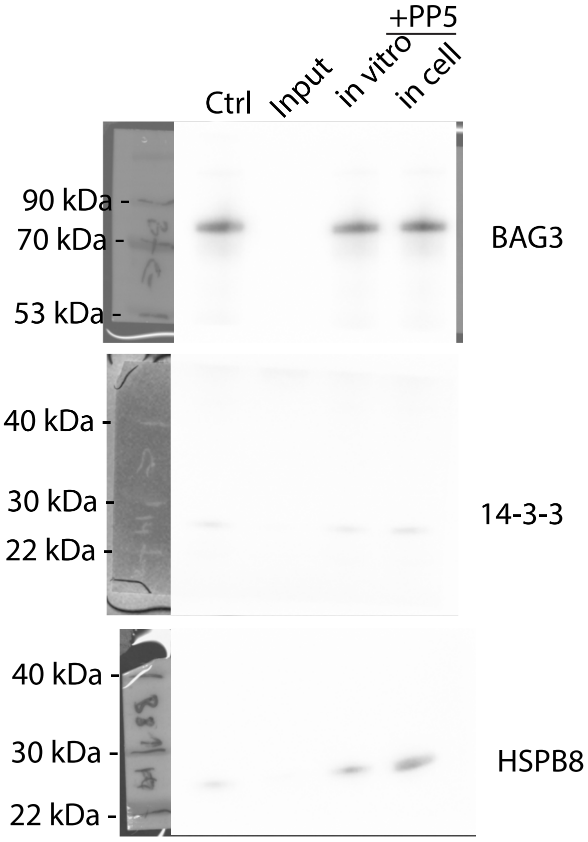
+PP5
Ctrl
Input
in vitro
in cell
90 kDa
70 kDa
53 kDa
BAG3
40 kDa
30 kDa
22 kDa
14-3-3
40 kDa
30 kDa
22 kDa
HSPB8

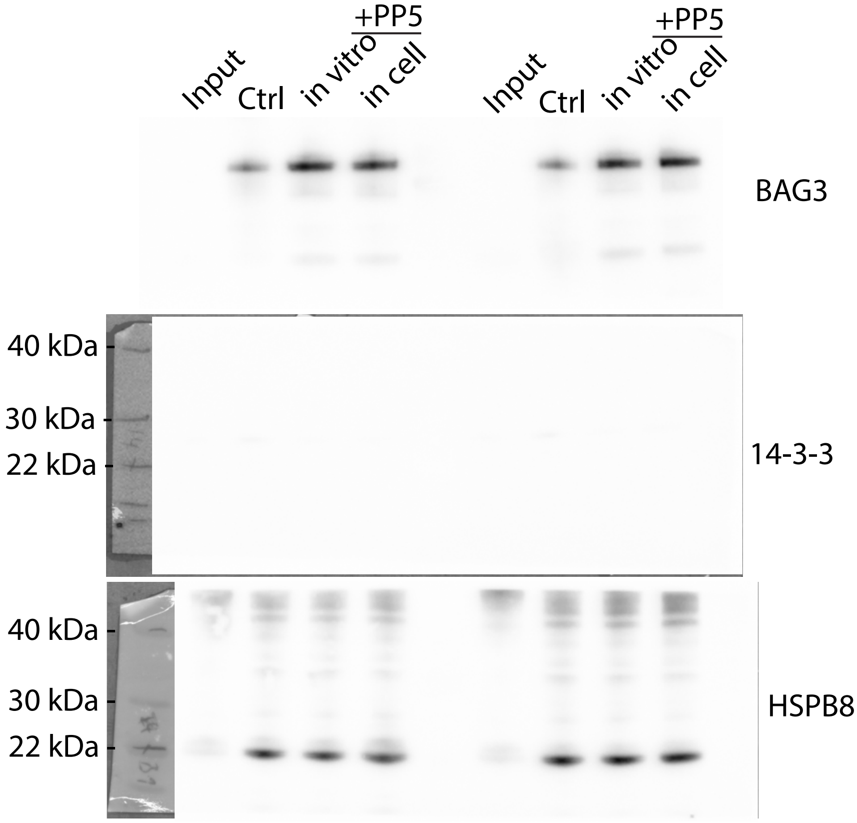
+PP5
+PP5
Input
Ctrl
in vitro
in cell
Input
Ctrl
in vitro
in cell
BAG3
40 kDa
30 kDa
22 kDa
14-3-3
40 kDa
30 kDa
22 kDa
HSPB8

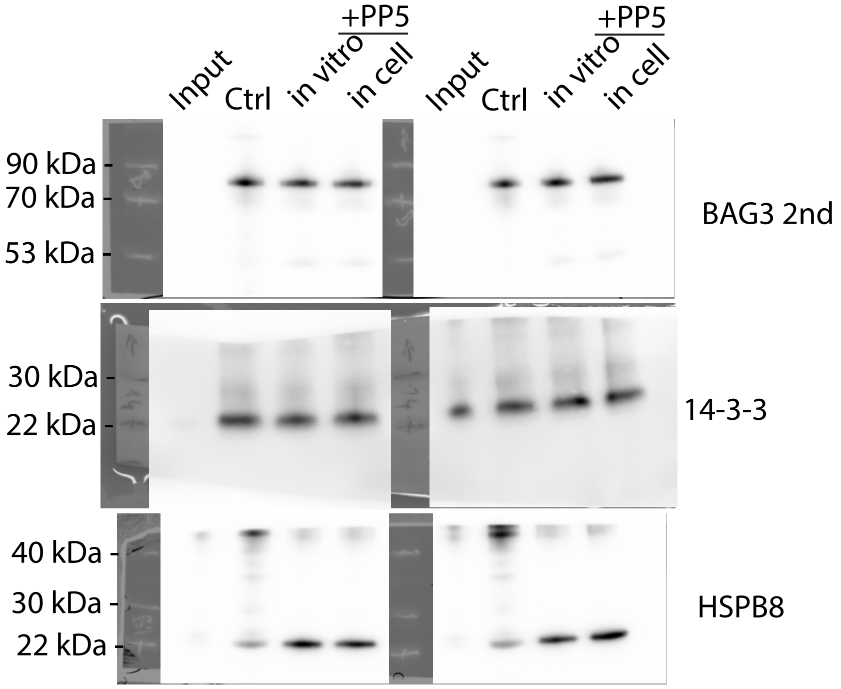
+PP5
Input
Ctrl
in vitro
in cell
+PP5
Input
Ctrl
in vitro
in cell
90 kDa
70 kDa
53 kDa
BAG3 2nd
30 kDa
22 kDa
14-3-3
40 kDa
30 kDa
22 kDa
HSPB8

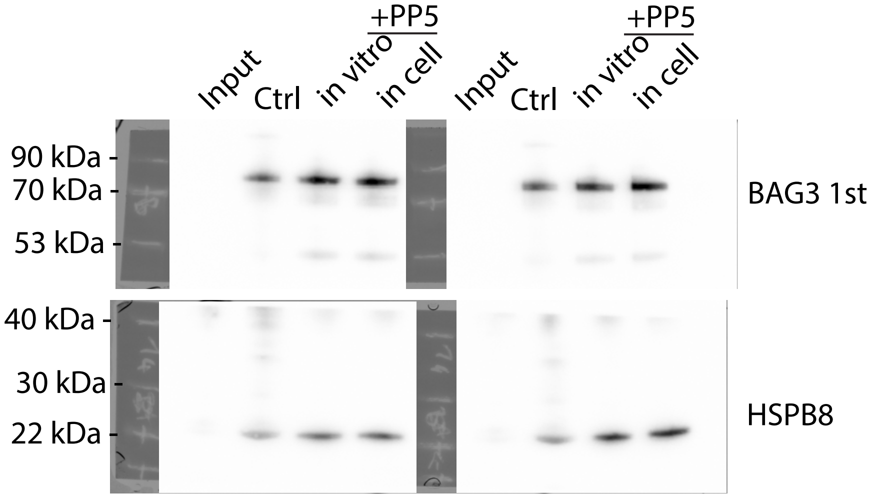
+PP5
Input
Ctrl
in vitro
in cell
+PP5
Input
Ctrl
in vitro
in cell
90 kDa
70 kDa
53 kDa
BAG3 1st
40 kDa
30 kDa
22 kDa
HSPB8

Figure 5F

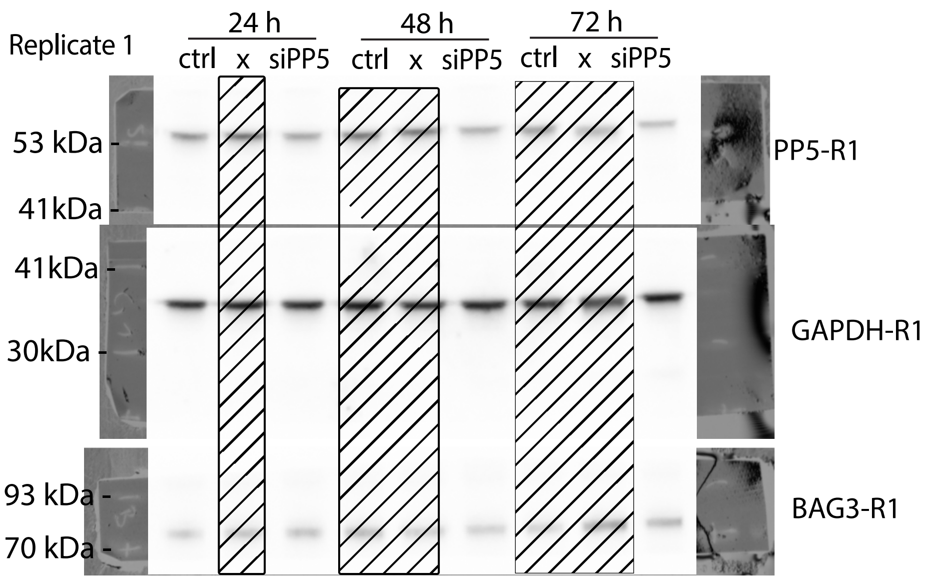

Replicate 1
24 h
48 h
72 h
ctrl x siPP5 ctrl x siPP5 ctrl x siPP5
53 kDa -
41kDa -
PP5-R1
41kDa -
30kDa -
GAPDH-R1
93 kDa -
70 kDa -
BAG3-R1

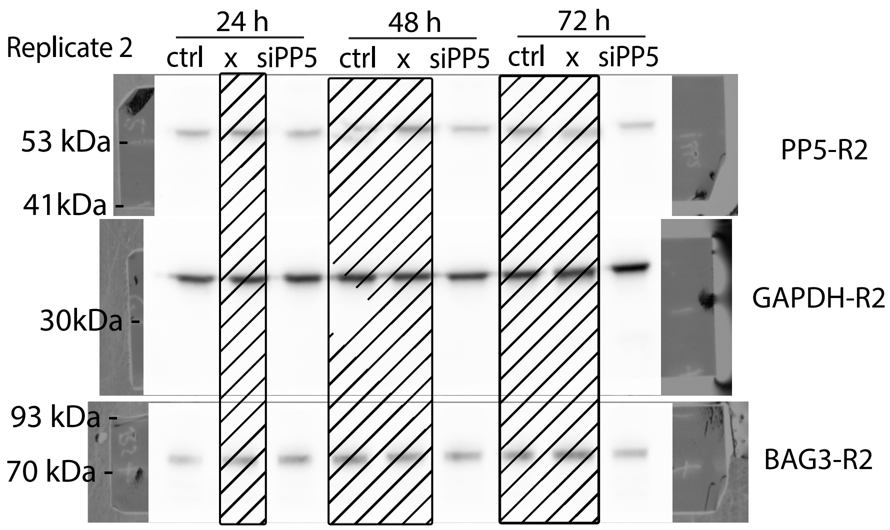

Replicate 2
24 h
48 h
72 h
ctrl x siPP5 ctrl x siPP5 ctrl x siPP5
53 kDa -
41kDa -
PP5-R2
30kDa -
GAPDH-R2
93 kDa -
70 kDa -
BAG3-R2

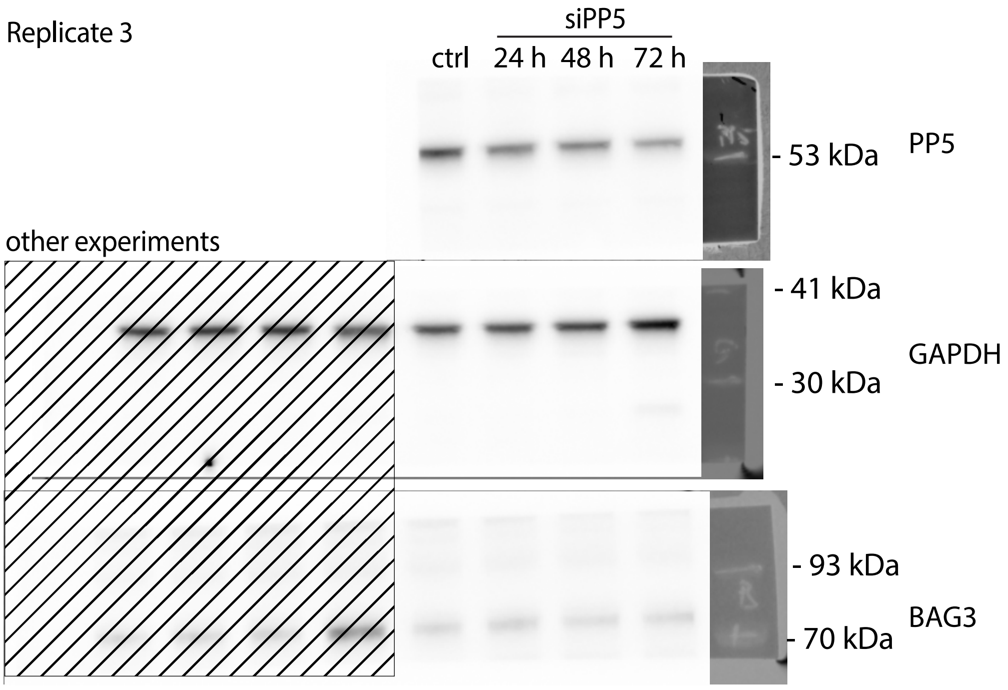

Replicate 3
siPP5
ctrl
24 h
48 h
72 h
- 53 kDa
PP5
other experiments
- 41 kDa
GAPDH
- 30 kDa
- 93 kDa
BAG3
- 70 kDa

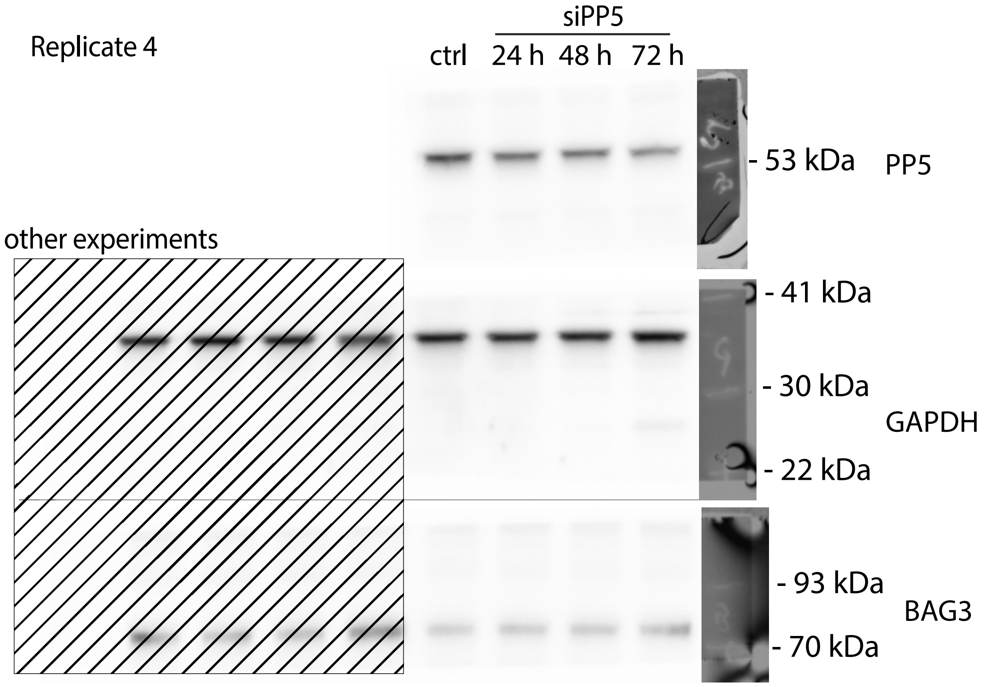

Replicate 4
siPP5
ctrl
24 h
48 h
72 h
- 53 kDa
PP5
other experiments
- 41 kDa
- 30 kDa
GAPDH
- 22 kDa
- 93 kDa
BAG3
- 70 kDa

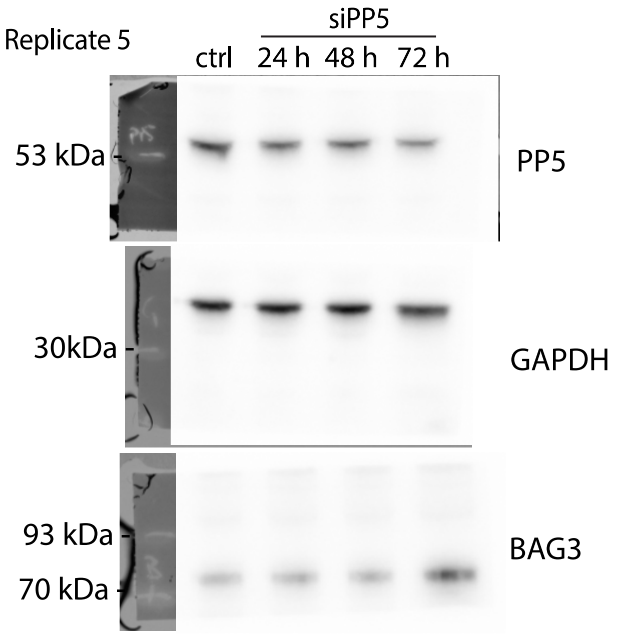

Replicate 5
siPP5
ctrl
24 h
48 h
72 h
53 kDa
PP5
30kDa
GAPDH
93 kDa
BAG3
70 kDa

Figure 5G/ Supplementary Figure 5F

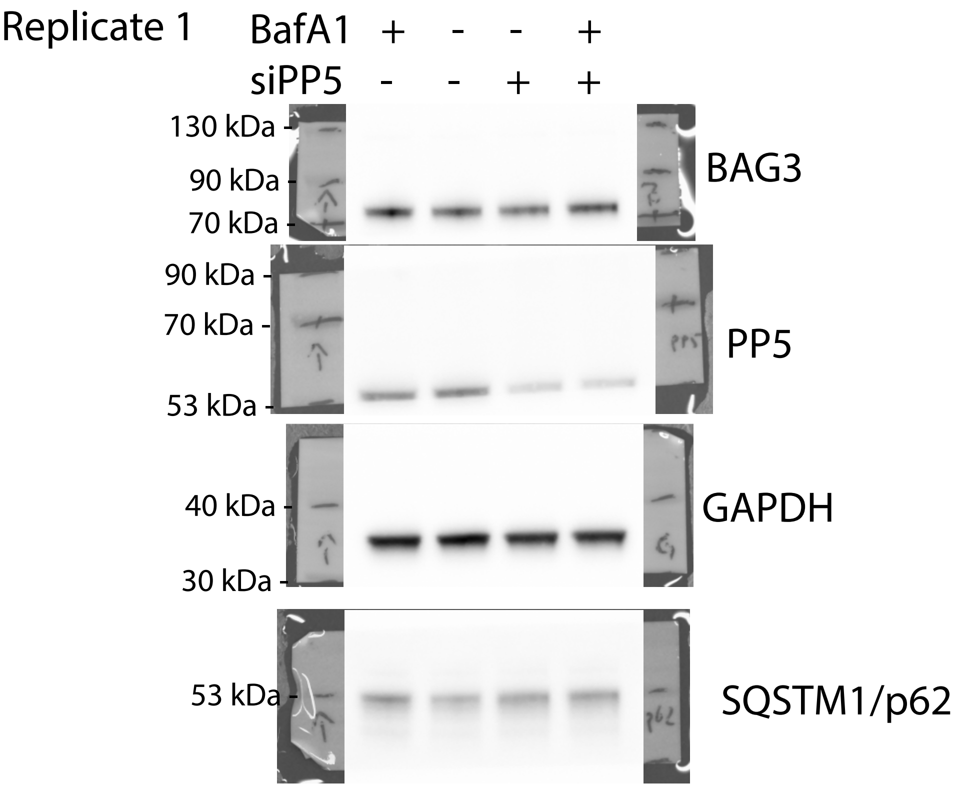

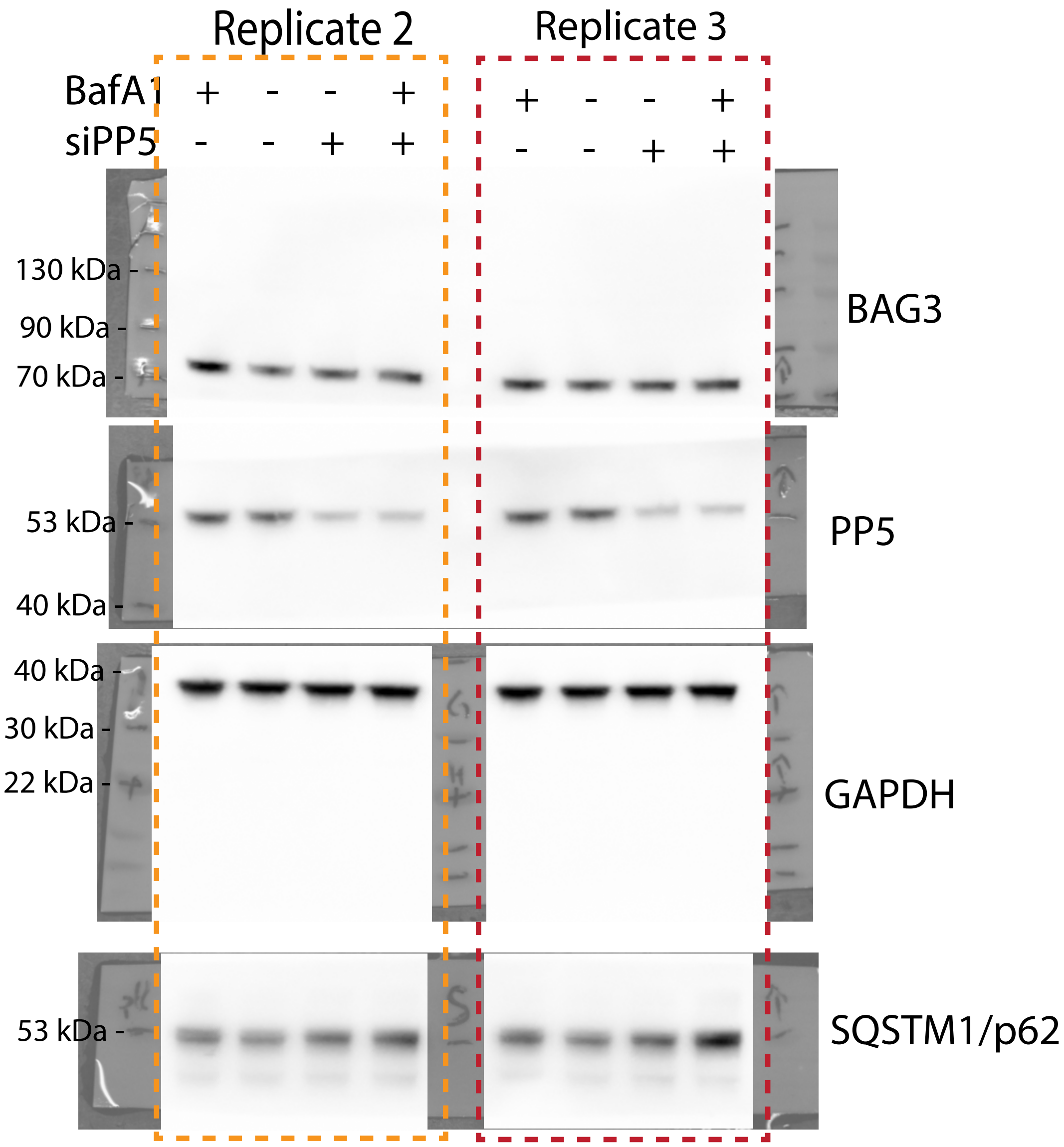

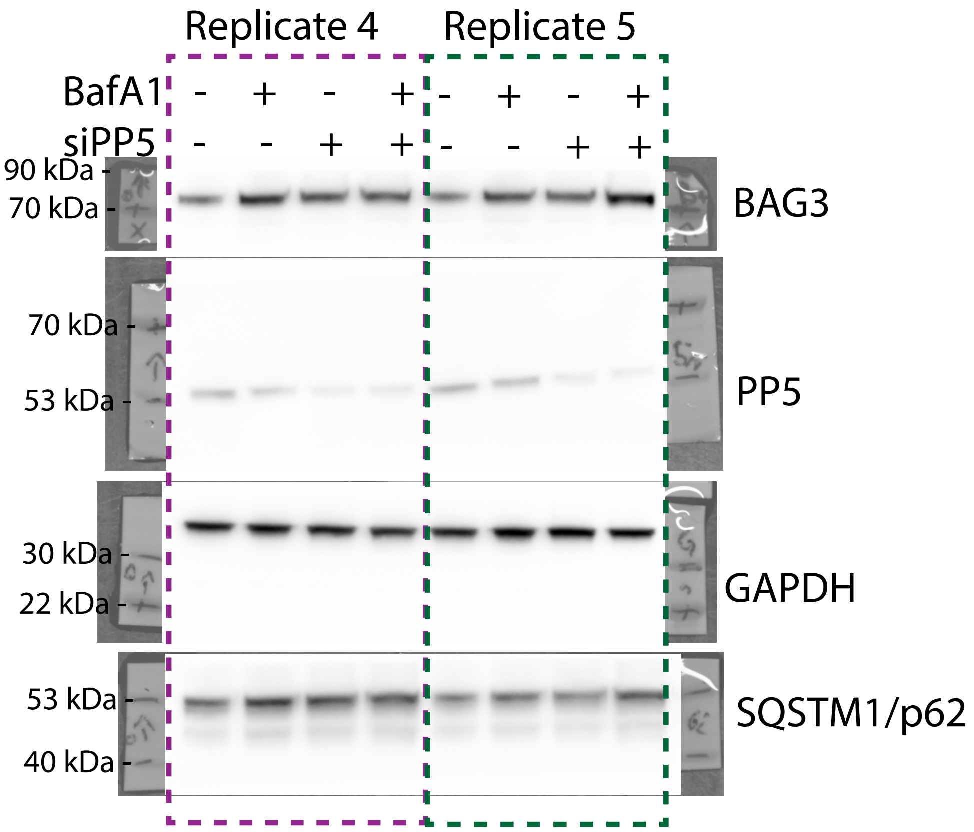
Replicate 4
Replicate 5
BafA1 - + - + - + - +
siPP5 - - + + - - + +
90 kDa
70 kDa
BAG3
70 kDa
53 kDa
PP5
30 kDa
22 kDa
GAPDH
53 kDa
40 kDa
SQSTM1/p62

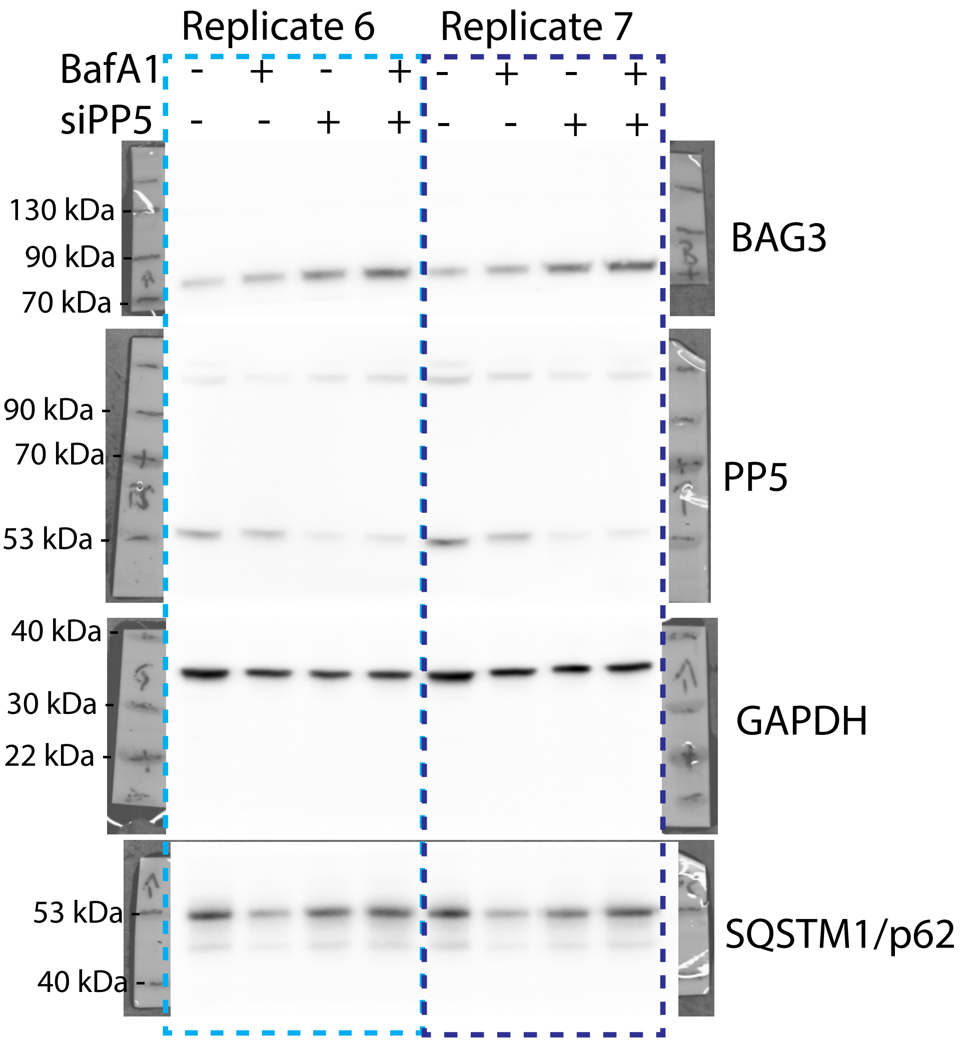
Replicate 6
Replicate 7
BafA1 - + - + - + - +
siPP5 - - + + - - + +
130 kDa
90 kDa
70 kDa
BAG3
90 kDa
70 kDa
53 kDa
PP5
40 kDa
30 kDa
22 kDa
GAPDH
53 kDa
40 kDa
SQSTM1/p62

Supplementary Figure 5C

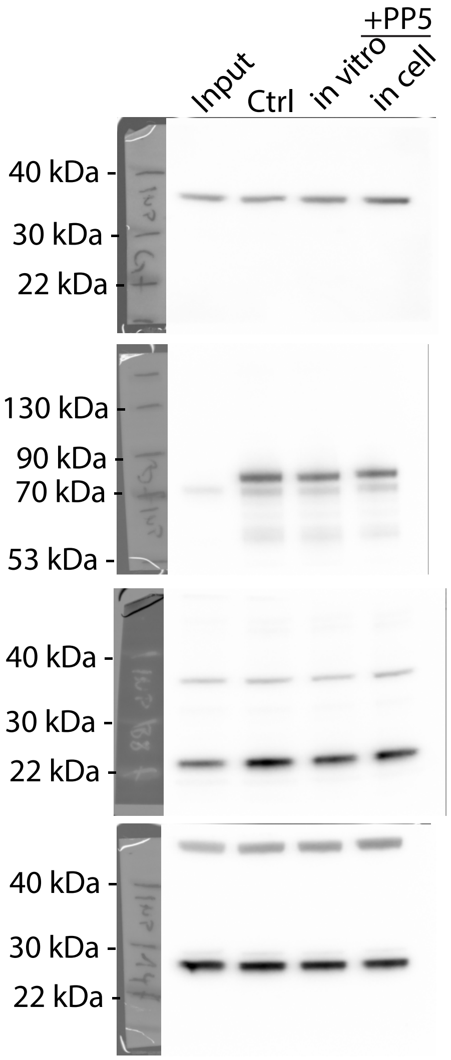

Supplement: Supplementary file 9 [file LSA-2024-02734_SdataF5.4_FS5.2.pdf]
